# High-Quality Graphene-Based Tunable Absorber Based on Double-Side Coupled-Cavity Effect

**DOI:** 10.3390/nano11112824

**Published:** 2021-10-24

**Authors:** Qiong Wang, Zhengbiao Ouyang, Mi Lin, Yaoxian Zheng

**Affiliations:** THz Technical Research Center of Shenzhen University, Shenzhen Key Laboratory of Micro-Nano Photonic Information Technology, Key Laboratory of Optoelectronic Devices and Systems of Ministry of Education and Guangdong Province, College of Physics and Optoelectronic Engineering, Shenzhen University, Shenzhen 518060, China; qwang@szu.edu.cn (Q.W.); linfengas111@szu.edu.cn (M.L.); jewel5282@163.com (Y.Z.)

**Keywords:** graphene-based device, tunable absorber, coupled-cavity system, finite difference time domain

## Abstract

Graphene-based devices have important applications attributed to their superior performance and flexible tunability in practice. In this paper, a new kind of absorber with monolayer graphene sandwiched between two layers of dielectric rings is proposed. Two peaks with almost complete absorption are realized. The mechanism is that the double-layer dielectric rings added to both sides of the graphene layer are equivalent to resonators, whose double-side coupled-cavity effect can make the incident electromagnetic wave highly localized in the upper and lower surfaces of graphene layer simultaneously, leading to significant enhancement in the absorption of graphene. Furthermore, the influence of geometrical parameters on absorption performance is investigated in detail. Also, the device can be actively manipulated after fabrication through varying the chemical potential of graphene. As a result, the frequency shifts of the two absorption peaks can reach as large as 2.82 THz/eV and 3.83 THz/eV, respectively. Such a device could be used as tunable absorbers and other functional devices, such as multichannel filters, chemical/biochemical modulators and sensors.

## 1. Introduction

Monolayer graphene, a semimetallic two-dimensional material of hexagonally arranged carbon atoms, has attracted significant attention due to its extraordinary mechanical, thermal, electronic, and optical properties. Similar to three-dimensional noble metal nanoparticles, monolayer graphene can also support surface plasmon-polaritons (SPPs), and it exhibits more remarkable properties such as flexible electrical tunability, strong light confinement, and relatively low ohmic losses [1,2,3,4]. Due to these unique characteristics, graphene-based absorbers, optical modulators, logic processors, power splitters, optical filters, and biochemical sensors have been realized [5,6,7,8,9,10,11,12,13].

Recently, great attention has been devoted to graphene-based absorbers. However, the absorption of monolayer graphene is very low. In order to solve this problem, some strategies have been proposed. For instance, high-efficiency absorbers have been realized by designing graphene patterns with different shapes, such as split ring, fishing-net and E-shaped models [14,15]. Another method is to use periodic metal units to excite surface plasmon polaritons to enhance the absorption of graphene [16,17,18,19,20,21]. In addition, periodic multi-layer dielectric materials have also been reported to enhance the absorption of graphene based on optical resonance effect [22,23,24,25,26]. These achievements open up a new way for designing graphene-based absorbers. However, in these approaches, no matter if adding periodic metal units or multi-layer dielectric materials to monolayer graphene, it needs a complicated fabrication process or it may give rise to a big size of device. Moreover, in these studies, only single-side coupled-cavity effect has been applied. These would bring limitations to applications. Therefore, graphene-based absorbers with high-efficiency and ultra-compact structure are necessary to be further explored.

In this paper, in order to improve the absorption of monolayer graphene and simultaneously keep the size of device compact, a new kind of absorber with monolayer graphene sandwiched between two layers of dielectric rings is proposed. The double-layer dielectric rings equivalent to resonators that are symmetrically added to both sides of monolayer graphene. Due to the double-side coupled-cavity effect, almost complete absorption is obtained. The influence of geometrical parameters on absorption performance is further discussed in detail. Also, the result demonstrates that a wide frequency range of absorption spectrum could be tuned by varying the chemical potential of graphene. Such a device could be used as tunable absorbers and other functional devices, such as multichannel filters, chemical/biochemical modulators and sensors.

Here, there are two points need to be emphasized. Firstly, different from some research in which only single-side coupled-cavity structures have been investigated [27,28,29], the double-layer dielectric rings we proposed to add to both sides of monolayer graphene could be more efficient to enhance the electric-field intensities on the upper and lower surfaces of graphene, whose double-side coupled-cavity effect could increase the absorption of graphene. Secondly, the double-layer dielectric rings do not require fabrication of metallic nanostructures [30] and could make the size of device more compact, compared with the periodic metal units or multi-layer dielectric materials reported in the other articles. The use of dielectric rings shows advantages in terms of fabrication cost since they have a lower price.

## 2. Structure Design and Calculation Method

**Figure 1:**
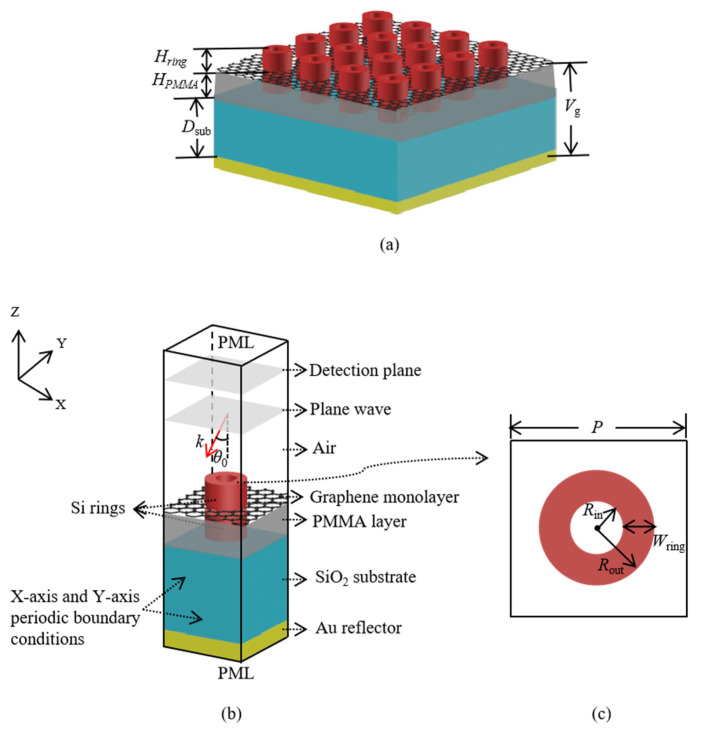
(**a**) The schematic of three-dimensional graphene-based absorber with monolayer graphene sandwiched between two layers of dielectric rings; (**b**) the simulation model of a pair of dielectric rings; (**c**) the x-y cross section of a single dielectric ring.

The schematic of graphene-based absorber we proposed is shown in Figure 1a. On an insulator substrate with refractive index *n*_sub_ = 1.45, monolayer graphene is sandwiched between two layers of identical dielectric-ring arrays. The dielectric is chosen as silicon with refractive index *n*_silicon_ = 3.4. The upper and lower layers of dielectric-ring arrays are symmetric with respect to the graphene plane. The lower layers of dielectric-ring arrays are embedded in polymethyl methacrylate (PMMA) layer with refractive index *n*_PMMA_ = 1.48. The thickness of the insulator substrate is set as *D_sub_* = 12 μm. A gold layer is deposited at the back side of the insulator substrate to present any transmission through the structure. In this way, perfect absorption can be obtained when the reflection is close to zero. The structure is periodic in the *x*-axis and the *y*-axis directions with periodic constant *P* = 8.5 μm. Figure 1b shows the simulation model of a pair of dielectric rings. Figure 1c shows the *x*-*y* cross section of a single dielectric ring. The inner and outer radii of dielectric ring are assumed as *R_in_* = 1.4 μm and *R_out_* = 2.4 μm, respectively. The height is set as *H_ring_* = 3 μm. The PMMA layer has the same thickness *H_PMMA_* = *H_ring_ =* 3 μm. For convenience in the following analysis, a new parameter *W_ring_* = *R_out_ − R_in_* standing for the width of the dielectric rings is defined.

For the structure, monolayer graphene is modeled as a conductive surface with thickness *D_g_* = 0.35 nm in the simulations. The surface conductivity *σ*(*ω*, *μ_c_*, Γ, *T*) in THz range can be expressed by the Drude model according to the Kubo formula, as given by [15],

(1) $$\sigma (\omega ,\mu_{c},\Gamma ,T)=\frac{e^{2}\kappa_{B}T\tau }{\pi \hbar^{2}(1+j\omega \tau )}\left\{\frac{\mu_{c}}{\kappa_{B}T}\right.\left.+2In\left[\exp(-\frac{\mu_{c}}{\kappa_{B}T})+1\right]\right\}$$

where *ω* is the radian frequency, *μ_c_* is the chemical potential of graphene, Γ *=* (*2τ*)^−1^ is the phenomenological scattering rate with *τ* standing for the relaxation time due to charge carrier scattering, *T* is the temperature, *ħ* is the reduced Plank constant, *κ_B_* is the Boltzmann constant, *e* is the electron charge. The relaxation time satisfies the relationship *τ* = *µµ*_c_/eν_F_^2^. Here, ν_F_ = 10^6^ m/s represents the Fermi velocity and *µ* is the carrier mobility of graphene. According to reported result, *µ* can be changed and the value as high as 4 m^2^/Vs can be obtained at room temperature [31]. Different *µ*_c_ is obtained by changing the dc voltage bias.

In the calculation, the finite difference time domain (FDTD) method is employed to calculate the reflection properties and simulate the electromagnetic field distributions of structure. As shown in Figure 1b, periodic boundary conditions are applied in both *x*-axis and *y*-axis directions to simulate the periodic structure. Perfectly matched layers (PMLs) are used at the outside of the top and bottom planes of calculation space to absorb the electromagnetic wave. In *x*-*z* plane, *θ*_0_ represents incident angle. When the incident electromagnetic wave reaches the gold layer, it is completely reflected if the thickness of gold substrate is set large enough. Therefore, the transmission is zero. The absorption of structure is calculated by *A* = 1 − *R* with *R* = *P_re_*/*P_in_*, in which *P_in_* and *P_re_* stand for the input and reflection energy flows, respectively. *P_re_* is detected at the back of incident wave plane.

## 3. Results and Discussions

**Figure 2:**
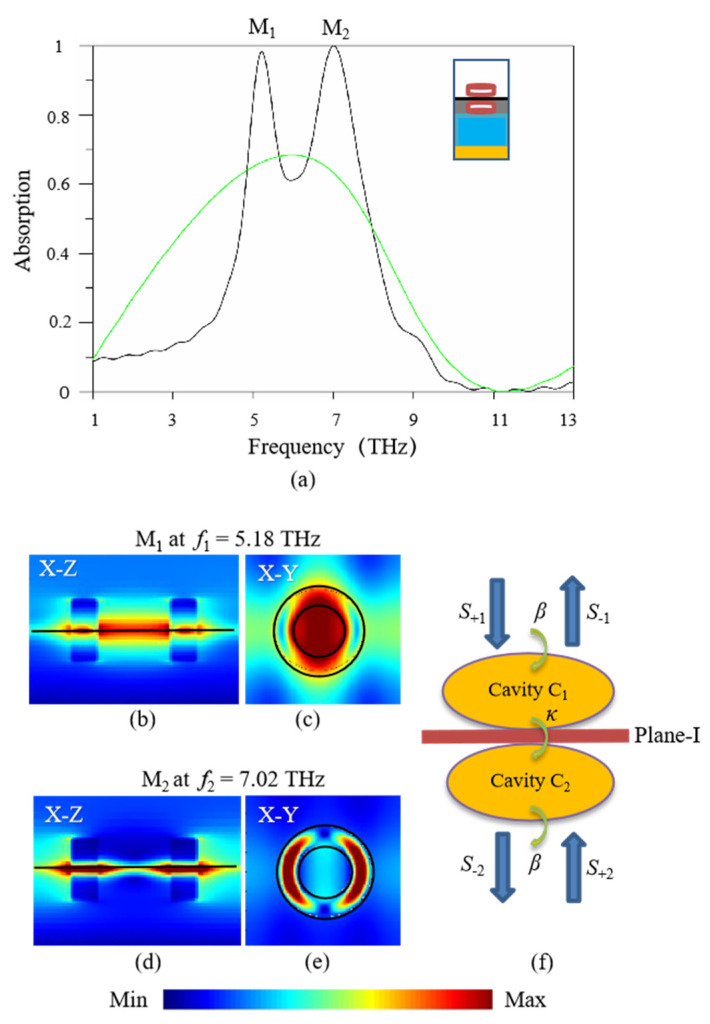
(**a**) The absorption spectrum of the proposed absorber with monolayer graphene sandwiched between two layers of dielectric rings, black line for *τ =* 1 ps and green line for *τ =* 0.05 ps, and its electric-field amplitude distributions at the absorption peaks for *τ =* 1 ps, (**b**,**c**) for mode M_1_ at *f*_1_ = 5.18 THz, (**d**,**e**) for mode M_2_ at *f*_2_ = 7.02 THz, and (**f**) the equivalent double-side coupled-cavity model.

In Figure 2a, it gives the absorption spectrum of the proposed graphene-based structure, as shown by the black line. For the parameters, *T* = 300 K, *τ =* 1 ps, *μ_c_* = 1.0 eV and *μ* = 1 m^2^/Vs are chosen. The period unit for calculating is given in the inset. Here, Under TE-polarized normal incident wave (TE wave, the electric field along *x*-axis direction), it can be seen that there are two obvious absorption peaks at *f*_1_ = 5.18 THz and *f*_2_ = 7.02 THz. For convenience, they are denoted as modes M_1_ and M_2_, respectively. The two peaks exhibit ultra-high absorption, reaching to 98.6% and 99.8%, respectively. In order to explore this absorption phenomenon, the electric-field amplitude distributions corresponding to the two absorption peaks are simulated and shown in Figure 2b–e. Figure 2b,c represent the electric-field amplitude distributions of mode M_1_ at the *x*-*z* plane (*y* = 0) and *x*-*y* plane (*z* = 0), respectively, and Figure 2d,e represent that of mode M_2_. It can be observed that the electric-field amplitude distributions of modes M_1_ and M_2_ are highly localized at the interfaces of the upper-layer dielectric rings and graphene layer, and the lower-layer dielectric rings and graphene layer. Not only that, there are also strong electric-field amplitude distributions localized in the inner space of the double-layer dielectric rings. As shown in Figure 2f, the double-layer dielectric rings added on both sides of the graphene layer are equivalent to double-side coupled-cavity model. For modes M_1_ and M_2_, the electric-field intensities of the upper and lower graphene interfaces are simultaneously enhanced by the resonance of double-side coupled cavities, which reinforces the graphene-wave interaction and enhances the absorption of graphene layer. On the other hand, referring to the literature [32], when the relaxation time of graphene is reduced to *τ =* 0.05 ps (*μ_c_* = 0.5 eV), the absorption spectrum is also calculated, as shown by the green line in Figure 2a. We can see that the absorption is reduced due to the decrease in the corresponding conductivity of graphene.

In order to further explain the numerical results above, the absorption performance based on the double-side coupled-cavity effect is also investigated by coupled mode theory (CMT) [23]. According to the simulation model of a single-period of the proposed graphene-based structure in Figure 1b, the upper-layer and lower-layer dielectric rings are respectively denoted as cavity C_1_ and cavity C_2_, and the unit cell of graphene layer is denoted as plane-I. Cavity C_1_ and cavity C_2_ are symmetric related to plane-I. The model of plane-I coupled with cavity C_1_ and cavity C_2_ can be considered as a coupled-cavity system, as shown in Figure 2f, which also can be described by the following formula based on the CMT analysis [33,34].

(2) $$\frac{da_{1}}{dt}=(j\omega_{1}-\beta_{1})a_{1}+\sqrt{2\beta_{1}}S_{+1}-j\kappa a_{2}$$

(3) $$\frac{da_{2}}{dt}=(j\omega_{2}-\beta_{2})a_{2}+\sqrt{2\beta_{2}}S_{+2}-j\kappa a_{1}$$

(4) $$S_{-1}=-S_{+1}+j\sqrt{2\beta_{1}}a_{1}$$

where *a*_i_ (*i* = 1, 2) is the mode amplitude of cavity C*_i_*, *ω_i_* (*i* = 1, 2) is the resonance frequency of cavity C*_i_*, *S*_+*i*_ and *S*_‒i_ are the amplitudes of the incoming and the outgoing waves for cavity C*_i_*, respectively, and *κ* is the mutual coupling coefficient between the two cavities C_1_ and C_2_, *β_i_* is the coupling coefficient between cavity C*_i_* and free space. We have *ω*_1_ = *ω*_2_ and *β*_1_ = *β*_2_ if considering the symmetry in structure, so new symbols *ω*_0_ = *ω*_1_ = *ω*_2_ and *β* = *β*_1_ = *β*_2_ are defined. In our case, it has *S*_+2_ = 0. Assuming that *S*_±*i*_ and *a*_i_ (*i* = 1, 2) have time dependence exp(*jωt*), we can obtain the following relation from Equation (3):

(5) $$\frac{a_{2}}{a_{1}}=\frac{-j\kappa }{j\omega -j\omega_{0}+\beta }$$

From Equations (2), (4) and (5), the absorption of structure can be calculated as:

(6) $$A=1-{\left|\frac{S_{-1}}{S_{+1}}\right|}^{2}=1-{\left|\frac{{\left(j\omega -j\omega_{0}+\beta \right)}^{2}+\kappa^{2}-2\beta \kappa }{{\left(j\omega -j\omega_{0}+\beta \right)}^{2}+\kappa^{2}}\right|}^{2}$$

As can be observed from Equation (6), at resonance frequency (*ω* = *ω*_0_), if *β* is equal to *κ*, perfect absorption occurs. This critical coupling condition suggests that the impedance matching between cavities C_1_ and C_2_ must be equal to that between cavity C_1_ (or cavity C_2_) and free space. When the incident wave is chosen at the resonance frequency (*ω* = *ω*_0_) and the system satisfies critical coupling condition (*β* = *κ*), resonant modes are excited at the interface of the upper-layer dielectric ring and graphene layer (called upper-layer resonant mode), and simultaneously excited at the interface of the lower-layer dielectric ring and graphene layer (called lower-layer resonant mode), as can be shown by the electric-field amplitude distributions of modes M_1_ and M_2_ in Figure 2b,d. The upper-layer and lower-layer resonant mode distributions are symmetric related to the graphene layer due to the high symmetry in structure. Based on the coupled-cavity effect, electric field intensity around the monolayer graphene is enhanced, which reinforces the graphene-wave interaction. As a result, the reflection wave of structure nearly vanishes and almost all the incident energies are absorbed. Perfect absorption occurs.

**Figure 3:**
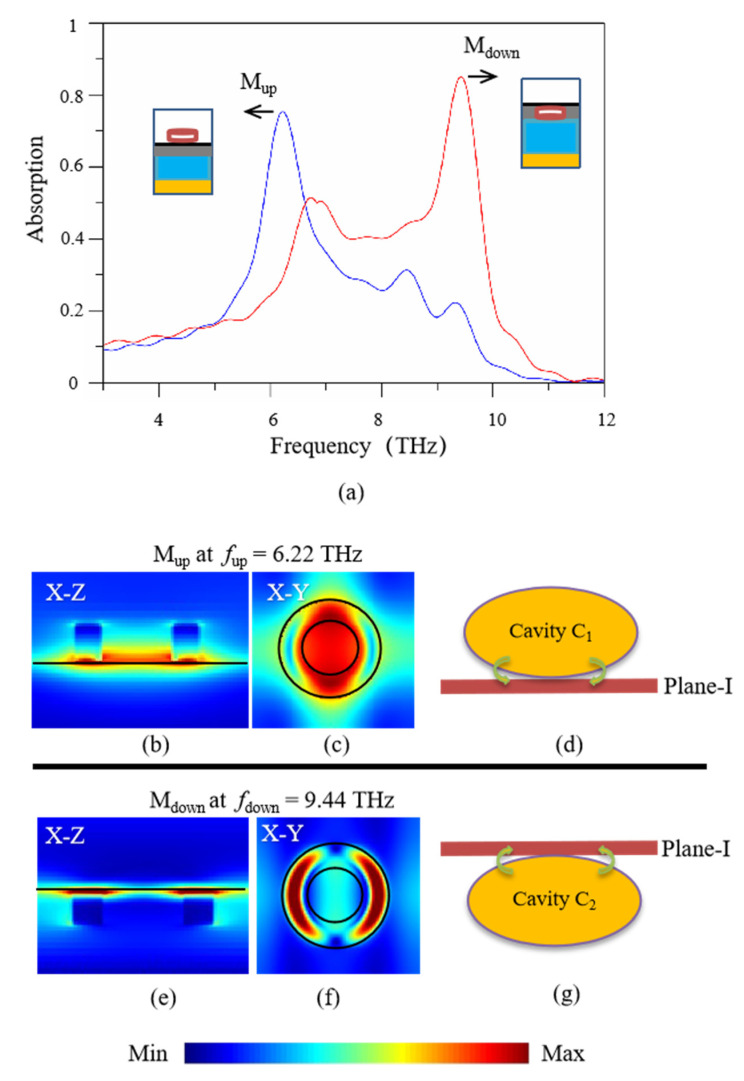
(**a**) The absorption spectra of simple structure 1 only with a single layer of dielectric rings added above the graphene layer (blue line) and simple structure 2 only with a single layer of dielectric rings added below the graphene layer (red line), and (**b**,**c**) the electric-field amplitude distributions at the absorption peak *M_up_* of simple structure 1 with *f_up_* = 6.22 THz, and (**d**) the equivalent single-cavity-above-graphene model; (**e**,**f**) the electric-field amplitude distributions at the absorption peak *M_down_* of simple structure 2 with *f_down_* = 9.44 THz, and (**g**) the equivalent single-cavity-below-graphene model.

For comparison, two relatively simple structures are considered, one is the structure only with the upper-layer dielectric rings (called simple structure 1), and the other is the structure only with the lower-layer dielectric rings (called simple structure 2). Their absorption spectra are plotted in Figure 3a, with the periodic units for calculating are given in the left and right insets, respectively. It shows that only one high-absorption peak appears for a single spectrum line, i.e., *f_up_* = 6.22 THz for simple structure 1 (see blue line) with absorption 75.5%, denoted as *M_up_*, and *f_down_* = 9.44 THz for simple structure 2 (see red line) with absorption 85.6%, denoted as *M_down_*. For mode *M_up_*, its electric-field amplitude distributions in Figure 3b,c show that there are strong field distributions at the interface of the upper-layer dielectric rings and graphene layer, and also at the inner space of the upper-layer dielectric rings. The upper-layer dielectric rings are equivalent to single-side resonance cavities added above the graphene layer, as shown in Figure 3d. On the other hand, for mode M_down_, its electric-field amplitude distributions in Figure 3e,f show that there are strong field distributions at the lower-layer dielectric rings and graphene layer. The lower-layer dielectric rings are equivalent to resonance cavities added below the graphene layer, as shown in Figure 3g. Comparing Figure 2 with Figure 3, we conclude that the absorption spectrum (black line) in Figure 2a is approximately a combination of the coupled effect of simple structures 1 (only with the upper-layer dielectric rings) and simple structures 2 (only with the lower-layer dielectric rings). The double-layer dielectric rings added on both sides of graphene layer can bring higher absorption efficiency than that of only adding a single layer of dielectric rings on one side of the graphene layer, which is because a double-side coupled-cavity model can yield stronger resonance effect than a single-side-cavity model.

In addition, the influence of the geometrical parameters (*W_ring_*, *H_ring_* in Figure 1) on the absorption performance are investigated. These analyses provide some guidance to structural design and fabrication.

**Figure 4:**
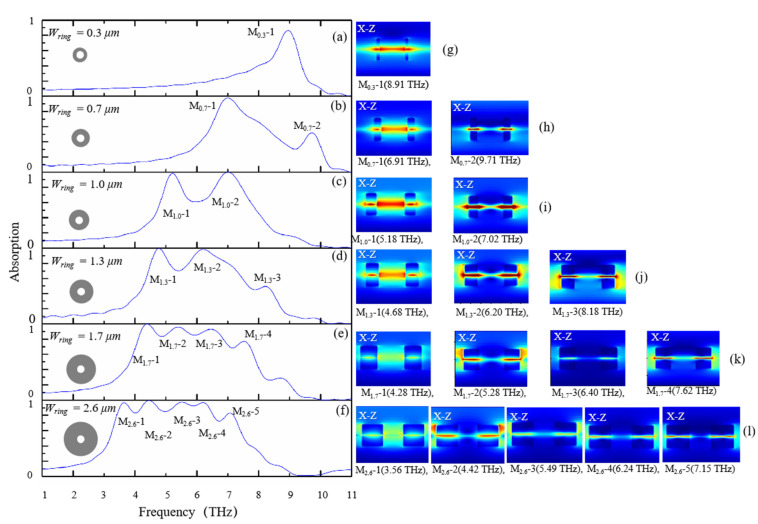
The absorption spectrum of the proposed graphene-based absorber when changing the width *W_ring_* of dielectric rings, (**a**) *W_ring_* = 0.3 μm, (**b**) *W_ring_* = 0.7 μm, (**c**) W*_ring_* = 1.0 μm, (**d**) *W_ring_* = 1.3 μm, (**e**) *W_ring_* = 1.7 μm, (**f**) *W_ring_* = 2.6 μm, and (**g**–**l**) standing for electric-field amplitude distributions of the absorption peaks.

First, we investigate the influence of the width *W_ring_* of the double-layer dielectric rings on the absorption performance. In Figure 4, it shows the change of absorption spectrum with the increase of *W_ring_*. We observe that when *W_ring_* is relatively small, such as *W_ring_* = 0.3 μm, there is only one absorption peak at 8.91 THz with absorption 87.7%. As *W_ring_* increases, two absorption peaks appear. It shows that when *W_ring_* = 1.0 μm, two absorption peaks appear at 5.18 THz with absorption 98.6%, and 7.02 THz with absorption 99.8%, respectively. When *W_ring_* further increases, the number of absorption peaks continues to increase. For instance, when *W_ring_* = 2.6 μm, five high-absorption peaks appear at 3.56 THz with absorption 95.5%, 4.42 THz with absorption 99.8%, 5.49 THz with absorption 95.5%, 6.24 THz with absorption 96.6%, and 7.15 THz with absorption 83.3%, respectively. For this case, the frequency range of absorption above 75% is from 3.35 THz to 7.22 THz, while that for the case of *W_ring_* = 0.3 μm is only from 8.69 THz to 9.14 THz. It means that the absorption band is greatly widened when *W_ring_* increases. This is because the relatively wide dielectric rings could bring an increase in contact area of the dielectric rings and graphene layer, then more resonance mode can be supported. The result also can be illustrated from the electric-field amplitude distributions of the absorption peaks for different *W_ring_*, as shown in Figure 4g–l. The characteristic provides a significant design guide for developing graphene-based absorbers with multiple resonance peaks.

**Figure 5:**
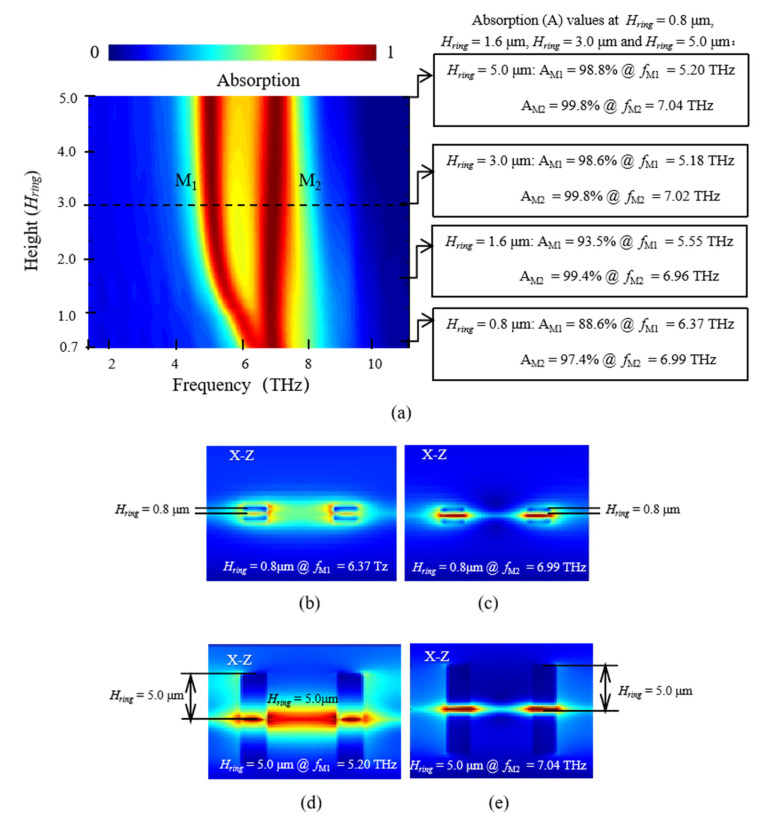
(**a**) The change of absorption of the proposed graphene-based absorber for different *H_ring_*, and the electric-field amplitude distributions of (**b**) *H_ring_* = 0.8 μm for M_1_, (**c**) *H_ring_* = 0.8 μm for M_2_, (**d**) *H_ring_* = 5.0 μm for M_1_, and (**e**) *H_ring_* = 5.0 μm for M_2_.

Next, the influence of the height *H_ring_* of the dielectric rings on the absorption performance is investigated. We chose the parameter *W_ring_* = 1.0 μm, referring to Figure 4c with two absorption peaks M_1.0_-1 and M_1.0_-2. Here, M_1.0_-1 and M_1.0_-2 are simplified as M_1_ and M_2_, respectively. As shown in Figure 5, when *H_ring_* is relatively small, such as *H_ring_* = 0.8 μm, the absorptions are 88.6% at 6.37 THz for the left mode M_1_, and 97.4% at 6.99 THz for the right mode M_2_. We can see that the absorptions are not high enough, which is because the dielectric rings with small height exhibit weak ability in z-direction spatial localization of electromagnetic wave (concluded from the comparison of Figure 5b–e), resulting in relatively weak resonance effect and unable to obtain enough strong absorption of graphene. When *H_ring_* further increases, the absorption peaks M_1_ has a red shift and the absorption peaks M_2_ has no obvious change in frequency. Their absorptions are gradually enhanced. When *H_ring_* ≥ 3.0 μm, the two absorption peaks tend to be stable not only in frequency position, but also in absorption amplitude. The positions are basically fixed at about 5.18 THz for M_1_, and about 7.02 THz for M_2_. Moreover, the absorptions are above 98.5% for M_1_, and above 99.7% for M_2_. Therefore, 3.0 μm ≤ *H_ring_* ≤ 5.0 μm is the best range since *H_ring_* being too small (*H_ring_* < 3.0 μm) is not of avail to the localization of electromagnetic wave, and an *H_ring_* too large (*H_ring_* > 5.0 μm) is not necessary for fabrication in practice.

**Figure 6:**
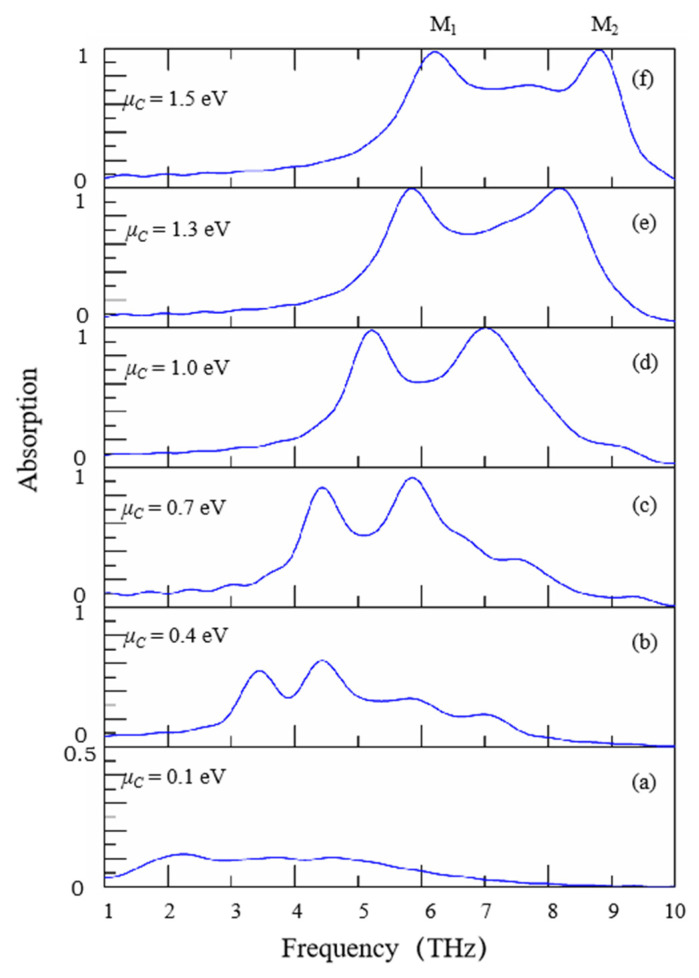
(**a**) The absorption spectra of the proposed graphene absorber for different *μ_c_*, (**a**) *μ_c_* = 0.1 eV, (**b**) *μ_c_* = 0.4 eV, (**c**) *μ_c_* = 0.7 eV, (**d**) *μ_c_* = 1.0 eV, (**e**) *μ_c_* = 1.3 eV and (**f**) *μ_c_* = 1.5 eV. *μ_c_* is the chemical potential of graphene.

On the other hand, one remarkable advantage of graphene is that its chemical potential *μ_c_* can be manipulated over a wide range through adjusting direct-current bias voltage *V_g_* that is added between the graphene layer and gold substrate. Due to this characteristic, graphene-based devices can be actively manipulated after final design and fabrication. As shown in Figure 6, it illustrates the influence of changing the chemical potential *μ_c_* of graphene on absorption performance. The parameters *W_ring_* = 1.0 μm, *H_ring_* = 3.0 μm are chosen, referring to Figure 4 and Figure 5. In this case, two absorption peaks M_1_ and M_2_ can be observed. In Figure 6, it shows that when *μ_c_* increases from 0.1 eV to 1.5 eV (*μ* = 1 m^2^/Vs), both of the two absorption peaks M_1_ and M_2_ exhibit obvious blue shifts and the frequency shifts can reach as large as 2.82 THz/eV for the left peak M_1_ and 3.83 THz/eV for the right peak M_2_, which demonstrates the remarkable tunability of structure. Meanwhile, when *μ_c_* increases from 0.1 eV to 1.5 eV, the absorption of the left peak M_1_ increases from 12.6% to 98.8%, and that of the right peak M_2_ also increases from 10.7% to 99.8%. It indicates that the absorptions of the two peaks are enhanced with the increase of *μ_c_*. Such a characteristic could be used as tunable absorbers and other functional devices, such as multichannel filters, chemical/biochemical modulators and sensors.

The frequency spectra of the absorption are represented in Figure 6 for different values of chemical potential obtained by increasing the dc voltage bias. The dielectric breakdown strength of the used materials is relatively high (large than 20 V/μm) in theory, which can satisfy the need of design. In practice, a layer of ionic gel would be applied over graphene layer. It can significantly reduce the voltage bias and avoid dielectric break down. This method has been demonstrated in the reference [35].

**Figure 7:**
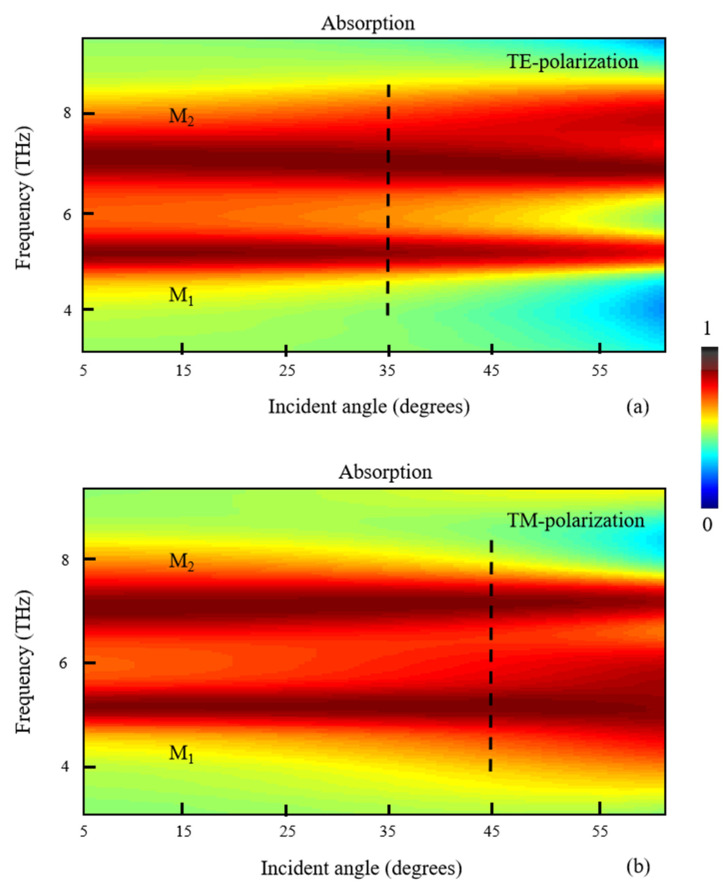
The effect of incident angle *θ*_0_ on absorption when the incident wave is chosen as (**a**) TE polarization and (**b**) TM polarization.

In the discussions above, absorption spectra have been investigated for normal incidence (*θ*_0_ = 0°). Finally, the absorption spectra at different *θ*_0_ are illustrated for TE and TM polarizations, as shown in Figure 7a,b, respectively. When the plane of incidence is set in the x-z plane, the incident angle *θ*_0_ is defined as the angle between the incident plane wave and the positive z-direction, as shown in Figure 1b. According to refraction theory, we have,

(7) $$n_{0}\sin\theta_{0}=n_{1}\sin\theta_{1}$$

where *n*_0_ is the refractive index of air, *n*_1_ is the effective refractive index of absorption domain and *θ*_1_ is the refraction angle. Here, *n*_1_ is complex due to the absorption. We can define *n*_1_ = *n*_1*r*_ + *i*·*n*_1*i*_, where *n*_1*r*_ and *n*_1*i*_ are the real and imaginary components of *n*_1_. Therefore, the refraction angle *θ*_1_ is also complex. It can be expressed as *θ*_1_ = *θ*_1*r*_ + *i*·*θ*_1*i*_, where *θ*_1*r*_ and *θ*_1*i*_ are the real and imaginary components of *θ*_1_. Therefore, the complex refraction angle *θ*_1_ is related to the parameters *θ*_0_, *n*_0_, *n*_1*r*_ and *n*_1*i*_. With FDTD Solutions software, the simulation model can be calculated by setting the parameters *θ*_0_, *n*_0_, *n*_1*r*_ and *n*_1*i*_, in which *n*_0_, *n*_1*r*_ and *n*_1*i*_ depend on the parameters of graphene and dielectric materials. It should be noted that the refracted plane wave into the absorber is nonuniform since it is closely related to the corresponding material in the refraction region. The absorption of structure can be indirectly calculated by collecting the total reflection energy flow at the detection plane, as shown in Figure 1b. In Figure 7, we can find that the absorption peaks M_1_ and M_2_ are almost unchanged at incident angles as large as 35° for TE polarization and 45° for TM polarization. This phenomenon is attributed to the high symmetry in the structure. The results would have important applications for wide-angle absorbers in practice.

## 4. Conclusions

In summary, we introduce a new kind of high-quality graphene-based absorber using a double-side coupled-cavity effect. In this structure, monolayer graphene is sandwiched between two layers of dielectric rings, which are equivalent to resonators coupled to both sides of graphene layer. The double-side coupled-cavity effect can greatly enhance the absorption of graphene. The result shows that two peaks with almost complete absorption are obtained. The influence of geometrical parameters on absorption performance are investigated in detail. Furthermore, through varying the chemical potential of graphene, the frequency shifts of the two absorption peaks can reach as large as 2.82 THz/eV and 3.83 THz/eV, respectively. The proposed structure also could be used for applications related to multichannel filters, chemical/biochemical modulators and sensors.

## Data Availability

The data presented in this study are available on request from the corresponding author.
